# Supplementary material for: Are carbon emissions trading and green financial instruments synergistic? -Comprehensive quantitative research based on content analysis
Source: PLoS One. 2024 Mar 7;19(3):e0298601. doi: 10.1371/journal.pone.0298601 (PMC10919601; doi:10.1371/journal.pone.0298601)
Supplement: S1 Data — (ZIP) [file pone.0298601.s001.zip › Data/地级市Z_F工作报告.zip!/║■▒▒/╧σ╤⌠╩╨/└┤╫╘╣1⁄2╓┌║┼í╛┬φ┐╦╩2╛▌═°í┐_2009.docx]

各位代表：

　　现在我代表市人民政府向大会作工作报告，请人大代表审议，并请政协委员和其他列席人员提出意见。

　　一、关于2008年的政府工作

　　过去的一年，在省委、省政府、市委的正确领导和市人大、市政协的监督支持下，市政府团结和带领全市人民，深入贯彻落实科学发展观，始终围绕跨越式发展目标，解放思想克难奋进，一门心思促进发展，努力克服特大冰冻、洪涝等多种自然灾害和国内外经济剧烈波动的重大影响，经济社会呈现出发展速度加快、质量提高、后劲增强、民生改善的良好态势。

　　（一）发展速度明显加快

　　经济总量加速扩张，初步核算，全市地区生产总值突破千亿元大关，达到1002.5亿元，三次产业构成由上年的17.8：43.6：38.6调整为17.5:45:37.5；全市地区生产总值、规模以上工业增加值、全社会固定资产投资和地方一般预算收入等主要经济指标分别增长14.6%、28.1%、40.6%和25.1%，均高于全省平均水平；尤其是地方一般预算收入突破30亿元，增幅创近五年新高。

　　工业经济实现“双千双百”新跨越。规模以上工业企业和产值“双过千”，规模以上工业企业净增307户，达到1023户；其中过10亿元企业净增9户，总数达到19户。规模以上工业产值突破千亿元大关，达到1082.4亿元，纺织、食品两大产业产值突破百亿元，汽车工业产值突破500亿元；完成规模以上工业增加值318.3亿元。

　　新农村建设取得新成效。各项惠农政策全面落实，促进了农村经济持续发展。建成高产农田22.9万亩，粮食总产达到413.1万吨，粮食和畜禽产品产量均创历史新高，烟叶、茶叶、水果、蔬菜等特色农产品生产水平进一步提升，新增国家级农业标准化示范区5个，认证有机、绿色、无公害农产品标识69个。农业产业化深入推进，农民专业合作社总量居全省第一位，116家市级以上龙头企业实现销售收入180亿元，同比增长25%。新农村建设试点村扩大到291个，13个村被评为首批“湖北省新农村建设示范村”。农村改革深化，集体林权制度改革确权发证面达到80%以上，农民专业合作社建设和城中村集体产权制度改革经验在全省推广。

　　国内外贸易繁荣活跃。全年完成出口额4.96亿美元，增长49%；市区中心商业区建设取得新进展，银泰百货等一批城市大型商业店场、农贸市场和农家店相继建成，促进了城乡消费；实现社会消费品零售总额410.3亿元,增长24.8%。

　　开发区建设和发展实现新突破。湖北深圳（襄樊）工业园和襄城、保康经济开发区获省政府批准设立，总投资15亿元的中国光彩事业襄樊工业园正式筹建，县市区一批特色工业园区开工建设，特别是湖北深圳（襄樊）工业园短短八个月就完成了一期工程的主要基础设施建设，18家企业达成入园协议，10个项目正式开工，一座现代化工业园区已具雏形。市县开发区完成投资161.7亿元，工业增加值237.9亿元，分别增长74.5%和43.5%。其中高新区完成投资80.2亿元，增长45.2%；规模以上工业增加值140亿元，增长29%；在全国54个高新区中的排名由上年的35位上升至34位。

　　县域经济发展势头强劲。省考核的7个县市区完成全社会固定资产投资197.6亿元，规模以上工业增加值112.5亿元，一般预算收入13.26亿元，分别增长61.6%、47.1%和26.5%，为近五年来最高增幅，分别高于全市21、19和1.4个百分点。南漳、保康一般预算收入突破亿元大关。

　　（二）发展后劲明显增强

　　完成全社会固定资产投资373.8亿元，增长40.6％，新开工项目1248个。完成制造业投资162.4亿元，增长63.8％，新天籁轿车等200个项目建成投产。100个亿元以上重大项目完成投资168.4亿元，29个项目竣工。

　　招商引资成效显著。全年签约招商项目1000多个，总投资1400多亿元。新增注册招商项目95个，投资总额233.8亿元，大唐电力、东湖高新、冠良实业、广东力天、三特索道、武汉凯迪等一批知名企业相继落户；新批外商投资企业22家，实际利用外资1.69亿美元，增长58.2%；同时，争取国家无偿投资11亿元，是上年的2倍。

　　金融支持发展力度加大。全市金融机构本外币贷款余额379.5亿元，比年初增加53.1亿元，贷款净增量居全省第二位；成功引进交通银行，争取建行、工行、交行授信268亿元。企业上市工作取得新进展，9家企业进入保荐程序，1家企业上市申请已由国家证券会正式受理。

　　（三）发展质量明显提高

　　创新能力进一步提升。争取实施国家和省科技项目72个；新增省级工程技术研究中心和企业技术中心7家；5个科技孵化器在孵企业195家；风险投资机制初步建立，襄樊创新资本创业投资有限公司正式运作；我市还相继成为国家知识产权试点城市和全省制造业信息化试点城市。创新人才队伍建设加强，建成博士后产业基地2个，引进硕士以上高层次人才145名。高新技术产业发展加快，新认定高新技术企业22家，完成高新技术产业增加值128.6亿元，增长21.5％。

　　节能减排成效显著。关闭小水泥企业3家，关停和搬迁改造市区化工企业11家，预计单位生产总值能耗下降4.8％，化学需氧量和二氧化硫排放量分别下降5.2％和1.5％；汉江水质继续保持二类标准。严格保护耕地，促进集约用地，实现了占补平衡。

　　（四）民生建设力度明显加大

　　市政府年初向全市人民承诺的十件实事全部完成，并于去年底将检查验收结果在媒体上进行了公示。

　　就业和社会保障进一步加强。培训转移农村劳力10.6万人，新增城镇就业9.3万人，其中安置城镇就业援助对象3070人，安置大中专毕业生1.2万人，城镇登记失业率控制在4.17%以内。新增社会保险参保人员18万人；城镇居民医疗保险制度全面实施，新型农村合作医疗进一步完善，337万农民和78万城镇居民享受到了有效的医疗保障；社会救助力度加大，提高了城区低保标准，新增农村低保人口1.35万人，新建、改建农村福利院18所，增加农村“五保”集中供养对象1011人，解决了农村1万多极端贫困人口的温饱和40多万人的饮水安全问题；住房保障措施加强，住房公积金制度进一步完善，改造农村危房3352户，新建城镇廉租住房5.84万平方米、经济适用房27.25万平方米，对市区人均住房建筑面积10平方米以下的困难家庭发放了租金补贴。城镇居民人均可支配收入和农民人均纯收入分别达到12292元和4880元，分别增长12.7%和18.6%。

　　公共事业实现新发展。义务教育进一步巩固，城区24所薄弱中小学改造全面启动，义务教育阶段农村学生书杂费和市区学生学杂费全部免除；对各学段贫困生的资助实现全覆盖；优质高中教育资源扩大，襄樊五中新校区建成投入使用，我市高考成绩居全省前列。加大了农村三级医疗保健网络、城市社区卫生服务体系和公共卫生服务体系建设力度，加强了中医药工作，有效控制了艾滋病、结核病、手足口病等重大传染病。人口出生率低于省定指标，符合政策生育率高于省定指标，出生人口性别比趋于合理。

　　应急管理加强，群众生命和财产安全保障力度加大。尤其在冰冻灾害和市区特大洪涝灾害等重大事件中，应急指挥、抢险救灾、急救防疫、灾民转移安置等措施及时有力，筹措救灾专款7000多万元，重建受灾家园，保障了15万受灾群众基本生活；汶川地震发生后，迅速组织全市上下捐资捐物，及时派出救援医疗队，扎实开展对口援建工作，目前我市已筹集援建资金和捐赠救灾物资总值5000多万元。安全生产和食品药品安全监管措施加强，安全生产事故死亡人数和受伤人数分别下降21.4%和48.5%。

　　（五）发展环境明显优化

　　政府效能建设扎实推进，“阳光新政”初见成效。以减程序、减时限、减收费为重点，深化审批制度改革，取消内外资工业项目申报资料217项，压缩工业项目审批时限70%以上，行政规费全部免除，服务效率显著提高。加强勤政廉政建设，推进政务公开，强化效能监察、审计监督和舆论监督，支持人大代表、政协委员视察经济发展环境，评议政府部门工作，严肃处理了4起行政不作为、乱作为案件及11名当事人，有力促进了政风好转。

　　基础设施改善，承载发展能力增强。市区基础设施建设完成投资18亿元，160个项目竣工投入使用，城市服务功能进一步提升。尤其是通过改造中心城区泥巴路、无灯街，新建停车场，延伸公交路线，更新公交车，实现中心城区供水管网和垃圾清运收集全覆盖，免费开放所有公厕，扩大城市绿地，改善了人居条件。国家卫生城市和国家环保模范城市创建工作扎实有效，城市管理水平明显提高。交通物流设施建设取得新进展，全面启动了市区内外环线建设，改造省际出口路、断头路和县乡公路370多公里，新建通村公路1400多公里；新建的汽车物流中心成为国内功能最强的轻卡物流园。

　　（六）民主法制和精神文明建设明显加强

　　依法治市深入推进。一年来，市政府自觉接受市人大及其常委会的法律监督和市政协的民主监督，认真办理人大代表议案、建议和政协委员提案；密切联系工会、共青团、妇联等人民团体，广泛听取民主党派、工商联、无党派人士的意见和建议，促进了民主决策、科学决策。完成了第三届城市社区居委会和第七届村委会换届选举工作，基层民主政治建设得到加强。大力推进依法行政，教育和引导政府执法机构强化依法行政、执法为民的理念，坚持处罚与教育相结合的原则，堵疏结合、宽严相济，初步形成了和谐、文明执法的新局面。信访工作进一步加强，一批群众反映强烈的信访问题得到有效解决，促进了社会稳定。以奥运安保为主线，强化社会治安综合治理，严厉打击各类犯罪，人民群众安全感进一步增强。

　　全国文明城市创建工作取得新成效，城市文明程度、行业文明水平和农村精神文明建设水平明显提高。建成了一批规范化“农家书屋”、乡镇文化站和农村体育活动场所，送戏下乡、送电影下乡活动广泛开展，以迎奥运为主题的各种大型文化体育活动精彩纷呈，丰富了城乡居民的精神文化生活。我市荣获“全省文明城市”称号，被中央文明委授予“全国创建文明城市工作先进城市”称号。驻襄解放军、武警官兵和民兵预备役人员积极投身地方经济社会建设，出色地完成了抗击冰冻灾害、抗洪抢险和入川抗震救灾任务，我市荣获“全国双拥模范城”称号。

　　各位代表，过去一年，全市经济社会发展取得的成绩，是省委、省政府和市委正确领导，市人大、市政协监督支持，全市人民团结拼搏、扎实苦干、奋发进取的结果。我代表市政府，向全市人民，向各位代表、各位委员，向各民主党派、工商联、人民团体和驻市单位，向驻襄人民解放军、武警官兵，向所有关心支持襄樊发展的同志们、朋友们，表示衷心的感谢，并致以崇高的敬意！

　　尽管过去一年的政府工作取得了一定的成绩，经济社会发展保持了良好的态势，但是我们也清醒地看到，经济社会发展面临的形势十分严峻：全球金融危机日益加深，导致外需下降，内需趋紧，尤其是与我市密切相关的汽车、纺织、电力等行业景气度明显下降，发展压力明显加大；经济结构不够合理，抵御市场风险的能力不强；农业基础设施仍较薄弱，农产品加工业和农村经济组织发展水平与现代农业建设的要求相比仍然滞后；支撑发展的大项目不足，承接国内外产业转移的力度有待加强；经济下滑导致外出务工的城乡劳力返乡增多，城乡就业压力和居民增收难度加大；关系民生的公共事业发展不够，社会保障水平有待提高；少数政府部门工作人员作风不实、效率不高、服务不优的问题还比较突出，发展环境有待进一步改善。对于这些矛盾和问题，我们将采取积极有效的措施，认真加以解决。

　　二、关于2009年政府工作的目标和任务

　　当前，全球金融危机对经济社会的影响正逐步加深。面对前所未有的困难和挑战，弱者看风险，强者看机遇。我们既要充分认识严峻形势，增强忧患意识；更要从危机中看到难得的机遇，坚定战胜困难、加快发展的勇气和信心。从政策层面看，针对经济运行中的突出问题，党中央、国务院把保增长作为当前的首要任务，果断采取了力度空前的应对措施，决定实施积极的财政政策和适度宽松的货币政策，相继出台了总投资4万亿元的十项扩大内需、促进经济增长的措施，三十条金融支持经济发展的意见和九大产业振兴规划，营造了良好的产业和民生发展环境；同时，党的十七届三中全会对农村改革发展作出了新的重大部署，作为农业大市，我市迎来了重大发展契机；国家促进中部崛起力度进一步加大，省委、省政府出台了两年投资1.5万亿的经济振兴计划，同时确定建设鄂西文化生态旅游圈，强调继续重视和加强省域副中心城市建设，形成了更加有利于加快发展的“小气候”。从市场层面看，国家扩大内需措施对经济增长的拉动作用正逐步显现；外需严重萎缩，迫使大批以出口为主的沿海企业加速向成本较低、市场广阔的中西部地区转移，为我市扩大招商引资带来了难得的机遇。与此同时，近年来我市经济在不利因素明显增多的情况下，呈现了快速发展的好势头，广大群众想发展、各级干部思发展的热情空前高涨。这些都为我们克服当前国内外经济环境恶化的不利影响，加快发展步伐，创造了良好条件。面对新的形势，我们必须坚定必胜的信心，抢抓千载难逢的历史机遇，趋利避害，克难奋进，努力在战胜危机中实现跨越式发展。

　　2009年政府工作的总体思路是：以党的十七大和十七届三中全会精神为指导，深入落实科学发展观，积极应对国内外宏观环境的新变化，抢抓国家强力促进经济增长和东部产业调整升级的重大机遇，紧紧围绕跨越式发展目标，突出抓招商、抓项目，努力扩大投资规模，积极调整经济结构，推进“三个百亿”工程，加快建设现代农业；突出改善民生，加强社会建设，加快发展社会事业，着力构建和谐安定的新襄樊，保持又好又快发展的好势头。

　　按照上述思路，2009年全市经济社会发展的主要预期目标是：地区生产总值增长11%，规模以上工业增加值增长16%，全社会固定资产投资增长30%，社会消费品零售总额增长20%，直接利用外资增长15%，外贸出口增长13%，地方一般预算收入增长13%，城镇居民人均可支配收入和农民人均纯收入分别增长9%和7%；约束性目标是:城镇登记失业率控制在4.6%以内，符合政策生育率达到92%以上，单位生产总值能耗下降4.8%，化学需氧量和二氧化硫排放量分别下降1.5%和2.6%。为实现上述目标，我们将着力抓好六项重点工作。

　　（一）千方百计扩大投资，构建跨越式发展新支撑

　　深入落实市委关于“项目促进年”的重大部署，千方百计聚集大投资，建设大项目，推动经济社会大发展，力争全社会固定资产投资突破500亿元。

　　把争取国家大投资、建设大项目作为跨越式发展的重要支撑。抢抓近两年国家实施4万亿、湖北实施1.5万亿投资计划的重大机遇，超前做好项目前期工作，储备一批符合国家投资方向的重大项目。组建强有力的工作专班，争取一批项目列入国家和省投资计划。重点争取襄樊机场改造工程、麻竹高速公路（襄樊段）建设、堤防标准化整治、病险水库除险加固、农村饮水安全工程、粮食增产工程、低产田和低产林改造工程、引丹入樊和“一洞一井”工程等一批重大项目列入国家和省投资计划。突出抓好120个投资过亿元的项目建设，争取当年完成投资260亿元以上；大力推进大唐集团新集电站等19个投资过10亿元的重大项目建设，为经济社会发展注入强大动力。

　　把招商引资作为推进跨越式发展的主要抓手。完善招商工作机制，赋予所有政府部门和直属机构招商引资职能，一个单位组建一个招商专班，深入发达地区驻点招商；将招商目标纳入政府工作目标考核，强化奖惩措施，促进全市上下形成全员招商的新局面。充分发挥发达地区襄樊商会、同乡会、外地在襄商会和外来投资企业的招商中介作用，拓宽招商渠道。进一步完善招商优惠政策，加大投资环境宣传推介力度，扩大襄樊知名度，增强招商吸引力。紧紧围绕做强产业链，培植新的经济增长点，争取在引进大项目上取得新进展，在引进新能源、新材料、新医药、节能环保、电子信息等新兴产业项目上取得新成效，力争每个县市区引进一、两个5亿元左右的投资项目；抓住国家推进军工企业整合的重大机遇，争取军工集团对我市军工企业实施战略重组，扩大战略投资，建设军民结合产业园；抓住“家电下乡”的契机，争取国内知名家电企业在襄樊建设辐射鄂豫陕渝毗邻地区市场的生产基地。力争全年新增工商注册招商项目400个以上，合同投资总额450亿元以上。

　　把扩大信贷规模作为推进跨越式发展的关键力量。抓住国家实施适度宽松货币政策的重大契机，策划一批优质项目，促进银企对接，争取金融机构全年贷款余额新增100亿元以上。以建投公司为平台，整合政府相关资产，增强融资功能，创新筹资方式，扩大融资规模；采取法人入股、财政注资、引进战略投资者等措施，促进城市商业银行增强实力；力争在引进股份制银行和非银行金融机构上实现新的突破，优化金融市场结构；推进农村金融产品和服务方式创新，抓好村镇银行等新型农村银行业试点工作，创办1-2家小额贷款公司，推广宜城“行业协会＋联保基金+银行”的融资模式，切实解决“三农”融资难。支持金融机构探索灵活多样的抵押方式，破解贷款抵押难题。着力打造“信用襄樊”，积极培植信用企业，优化金融生态环境，促进银企合作双赢。

　　把促进全民创业作为激活民间投资、推进跨越式发展的重大战略。全面落实支持全民创业的政策措施，促进全市民营经济发展大突破。从放宽准入条件、提供融资担保、强化财税扶持、减免行政规费、落实创业用地等方面，加大创业扶持力度。突出抓好担保体系建设，增强市中小企业担保中心和农业产业化担保中心的贷款担保能力，大力支持全民创业；实行以奖代补，扶持初始创业。依托市县开发区建设创业孵化园，鼓励民间投资创办各类创业基地，搭建优越的创业平台。充分发挥创业服务机构职能，设立创业服务热线，及时为创业者提供政策咨询、技能培训、融资辅导和用工信息等服务。大张旗鼓地评选表彰“十大创业明星”和民营企业50强，营造全社会全力支持创业、全民踊跃参与创业的良好氛围。力争全年新增个体私营等非公有制经济组织5000家以上。

　　把加强基础设施建设作为拉动投资增长、提升承载能力、促进跨越式发展的重要举措。着眼于提升城市功能，加大城市建设力度。坚持以规划为先导，着力打造“数字襄樊”，坚决遏制违法建设，促进城市科学建设。系统改造城市排水设施，基本解决市区内涝问题；实施一批路网工程，重点推进内外环建设，着力解决城市交通拥堵问题；抓好城市绿化、城中村改造、天然气管道延伸、城市污水处理和固废处理设施建设工程，在城市规划区内停批一户一宅，强力推进“村民公寓”建设，综合整治城市环境。积极创建国家卫生城市和环保模范城市，扎实做好国家园林城市复查迎检工作，严格执行城市管理法规，巩固城市管理成果，提高管理水平。抓好电力、交通和通讯设施建设。完成207国道和316国道襄樊段改线和140公里省际出口路、断头路和县乡公路建设，做好福银高速公路谷城至竹溪连接线和麻竹高速公路襄樊段的前期工作，争取尽快开工；支持襄樊机场改造，提升区域综合交通功能。支持第三代数字通信设施建设，确保第三代数字通信网络尽快开通。

　　（二）强力推进“百亿工程”，带动经济发展新跨越

　　坚定不移地把培育百亿产业、打造百亿企业、建设百亿园区作为推进新型工业化、支撑经济跨越式发展的主导力量。

　　着力促进百亿产业发展壮大。紧紧围绕汽车产业过千亿的目标，强化服务和推进措施，大力支持东风公司襄樊基地加快建设和发展，促进湖北福田和江山重工做大做强，重点抓好东风股份A08轻型客车、江山重工特种车等16个投资过亿元的汽车及零部件项目建设，促进车桥、轴承、摩擦材料等特色零部件集群发展，增强汽车产业竞争力。以完善产业链和提高技术装备水平为重点，着力振兴轻纺工业，重点建设冠良实业李宁运动鞋、力天鞋业工业园、宁波服装工业园等6个投资过亿元的新项目，拓展下游产业链。突破性发展食品工业，提高粮油、肉食精深加工水平，推进白酒行业整合，大力发展高附加值的农副食品加工业，着力抓好襄樊烟厂、奥星粮油、龙建集团、雨润集团等10个投资过亿元的项目建设。

　　加速培育新的支柱产业。科学把握国家产业政策新导向和市场需求新变化，把磷化工、新型建材、冶金和绿色能源作为新的百亿产业来培育。着力抓好泽东公司磷铵、龙蟒集团磷酸盐等6个投资过亿元的磷化工项目建设，在引进中化集团、兵器集团战略投资和争取兴发集团、天舜集团扩大投资上实现突破；支持葛洲坝集团新型干法水泥等4个投资过亿元的新型建材项目建设；尽快启动西部矿业铅锌冶炼和枣阳金红石矿开发，加快建设金洋公司再生铝、再生铅等3个投资过亿元的有色金属冶炼项目；大力推进汉江梯级开发，启动大唐集团新集水电站建设，做好雅口电站前期工作，确保崔家营航电枢纽第一台机组发电，抓好凯迪、盛华、安能生物质能源和骆驼、润阳环保电池等投资过亿元的新能源项目建设，壮大绿色能源产业。与此同时，大力发展农业机械、轨道交通设备、数控机床、螺旋杆空压机、矿山防爆发电机组和防爆车等特色装备制造业；突破性发展航空航天装备、电子电器、节能环保设备、新材料等特色高新技术产业。

　　深入实施企业成长工程。按照“做强一个龙头企业，带动一群中小企业，形成一个产业集群”的思路，大力推进企业成长工程。支持行业龙头企业以增强市场竞争力为重点，狠抓技改、狠抓大项目，促进上市公司做大做强，把争取股票上市、引进战略投资、实施并购扩张战略，作为促进龙头企业制度创新、管理创新、技术创新和跨越式发展的重要抓手，力争取得新的突破。以健全服务机制、完善服务体系为抓手，大力促进中小企业发展。以市场为纽带，建立中小企业与龙头企业合作发展促进机制，扩大中小企业发展空间；支持龙头企业整合行业资源，创办工业设计中心、印刷包装中心、模具中心、锻造中心和精密加工中心等公共基础平台，增强对中小企业的辐射带动能力；实行财政贴息，提供贷款担保，帮助中小企业解决“融资难”；集中使用科技“三项经费”等专项资金，扶持一批成长性好的中小企业加速发展。引导改制企业完善法人治理结构，建立现代企业制度，提高经营管理水平。全面落实国家结构性减税、行政规费减免等政策，支持企业积极应对经济波动，帮助困难企业扭亏解困，稳定发展。确保今年新增规模以上企业200户。

　　强力推进开发区建设。多渠道投资30亿元左右，加大各级开发区建设力度。促进高新区做大做强，昂起全市经济的龙头。加快建设高新区台商园“七通一平”、东湖高新襄樊电子园标准厂房和汽车园综合服务区；推进开发区制度创新和服务创新，加强科技孵化器、知识产权试点园、公共技术平台等服务体系建设，高水平策划、建设和宣传高新区品牌形象，提升软实力，增强要素吸引力；加快产业集聚步伐，高新区规模以上工业产值突破600亿元。围绕培植新的经济增长极，加快发展湖北深圳（襄樊）工业园，全面建成“七通一平”基础设施，启动建设中心商务区，提升园区承载功能；完成襄城经济开发区“五通一平”和樊城经济开发区广东轻纺工业园起步区基础设施建设，进一步完善樊西工业园基础设施。支持县市经济开发区增强产业集聚能力，使之真正成为县域工业发展的主要载体，促使襄阳、谷城、枣阳、老河口等县市区规模以上工业产值突破百亿元。

　　（三）加快建设现代农业，引领社会主义新农村新发展

　　深入贯彻落实党的十七届三中全会精神，以建设现代农业为突破口，推进农村改革和发展，努力开创新农村建设新局面。

　　大力推进“一县一品”，加快建设现代农业板块。紧紧围绕满足农产品加工业发展需要和市场需求，深入实施“一县一品”战略，按照一个县市区突出发展1-2个主导产业的要求，加快建设一批优质特色农业板块基地。以金华麦面集团、梨花湖食品公司等企业为龙头，着力建设300万亩优质专用小麦基地；以万宝粮油、梅园米业等企业为龙头，着力建设200万亩优质水稻基地；以奥星粮油、襄阳鲁花等企业为龙头，着力建设150万亩“双低油菜”、优质花生和芝麻等油料基地；以际华三五四二、银河纺织等企业为龙头，着力建设50万亩优质棉花基地；以祯德食品、丽波乳业等企业为龙头，着力建设一批规模化、标准化、生态化养殖小区，创建畜牧水产大县、大镇，规模养殖量达到40%以上；以大山现代农业公司、乾兴农业公司、光明集团茶叶公司等企业为龙头，着力建设150万亩无公害蔬菜、30万亩无公害茶叶、50万亩核桃等特色产业基地；以仙仙果品等企业为龙头，着力建设30万亩优质桃、梨等水果基地；以襄樊烟草公司为龙头，争取国家扶持发展现代烟草种植业；以武汉凯迪等企业为龙头，启动建设100万亩油茶基地和50万亩生物质能源林基地；以威利邦等企业为龙头，加快建设87万亩速生丰产林基地。

　　加强综合生产能力建设，夯实现代农业发展基础。全面落实国家惠农政策，争取国家和省更多支持，加大财政支农力度，引导农民增加农业投入。大力实施粮食增产工程，争取襄阳、枣阳、宜城、老河口进入国家千亿斤粮食增产计划，新建高产农田15万亩，粮食产量稳定在80亿斤左右。争取启动引丹“一洞一井”工程，抓好50座病险水库除险加固、5个大型灌区续建配套和节水改造工程，建设一批小型农田水利设施，修复一批水毁泵站、渠系，恢复扩大灌溉面积45万亩。适应食品安全新要求，着力建设全国知名的绿色食品生产基地。努力提高科学种养水平，培育一大批农业科技示范户，建设11个农业科技示范基地；建好鄂西北农作物种质资源库，培育、筛选和引进一批优质、高产、专用的动植物新品种；大力推广标准化生产、植保综合防治、标准化饲养等40项先进生产技术，创建4个省、市级农业标准示范区，新认证40个绿色、有机、无公害农产品，重点培育10个优质农产品品牌。扎实抓好国家级农业机械化示范区建设，大力推广农业机械新机具、新技术，力争农机综合作业水平提高到70%以上。加强动植物疫病防治和气象预测预报工作，充分做好防汛抗旱准备，增强农业抗灾、减灾、避灾能力。

　　推进体制机制创新，增强现代农业发展活力。深入贯彻落实中央关于加快推进农村改革与发展的决定，深化农村改革。建立健全土地承包经营权流转管理和服务体系，引导农民按照依法、自愿、有偿的原则，多种形式流转土地承包经营权，发展适度规模经营。深化集体林权制度改革，完成确权发证任务，规范和完善森林资源流转等系列配套改革。支持国有农牧渔良种场改革和发展，力争建成现代农业示范基地。大力发展村级集体经济，增强造血功能。创新农业经营方式，采取以奖代补、贷款贴息、优先安排国家投资项目等措施，促进农民专业合作社突破性发展，五年内，每年在抓好10个市级示范社建设的同时，每个县抓3个示范社、每个乡镇抓1个示范社建设，每年新增农民专业合作社100家以上。

　　加快城乡一体化步伐，提高新农村建设水平。统筹城乡规划建设，科学编制城镇和村庄建设规划。高度重视和加强乡镇建设，充分发挥其在城乡一体化发展中的纽带作用。深入实施“百镇千村”示范工程，新办200个村庄整治试点。继续抓好新农村建设试点工作，引进战略投资者，建设樊城区太平店、牛首和柿铺新农村示范片（带），逐步使之成为全市城乡一体化发展实验区、现代农业示范区和新农村建设先行区。

　　（四）积极培植消费热点，促进现代服务业发展新突破

　　做强旅游经济，拉动旅游消费。抢抓鄂西生态文化旅游圈建设机遇，突出“三国文化”和“荆山生态”两大主题，策划实施一批大型旅游开发项目，引进战略投资者，着力建设全国重要的文化生态旅游目的地、中西部重要的游客集散地，逐步建成鄂西生态文化旅游圈的核心区和支撑点。按照5A级景区标准，抓紧修编古隆中景区规划，启动一批景区基础设施和旅游项目建设。按照4A级景区标准，推进保康九路寨、南漳古山寨、谷城薤山（南河）、襄城回龙湖和枣阳白水寺等五大景区开发，力争两年内建成4个4A级景区。突出培植10个旅游明星镇和30个特色旅游村，支持南河创建湖北旅游名镇，打造生态旅游新亮点。围绕整合岘山、襄阳古城和汉江旅游资源，完成山水古城三位一体旅游发展规划编制；组建米芾书画院，着力打造全国著名的书画艺术交流和培训基地。加快报批鱼粱洲总体规划，力争启动生态旅游开发。加大媒体宣传推介力度，打造襄樊文化生态旅游品牌。强化旅游市场开拓，吸引国内龙头旅行社经营襄樊旅游，支持本地旅行社与旅游集散地城市旅行社联合开发组团游，与周边著名景区联合打造区域热点线路。延伸旅游服务链条，提高酒店和餐饮服务业水平，规划建设特色饮食服务街区；加快发展文化、体育、休闲、娱乐等消费产业。

　　促进国内外贸易，扩大地产品消费。积极创建樊城省级现代服务业示范区，抓好春天大道步行街、大明广场、华康保险全国培训中心等6个总投资过9亿元的项目建设，促进樊城现代服务业提档升级；继续实施“万村千乡”、农村“新网”和“双百市场”建设工程，支持超市进乡镇；推进“家电下乡”，扩大农村家电消费。鼓励信贷消费、促进农超对接，大力支持企业开拓国内外市场；认真落实国家房地产政策，规范房地产市场秩序，鼓励住房消费，促进房地产市场健康发展。用好用足出口优惠政策，优化出口环境，支持出口大户增强国际竞争力，鼓励一批发展前景好、出口潜力大的中小企业加快发展；鼓励企业争创精品名牌，增强市场开拓力，争取今年新增10个省级名牌和著名商标、2-4个中国名牌和中国驰名商标。

　　壮大现代物流业，扩大生产性消费。以建设全国区域性物流中心为目标，加快发展具有襄樊特色的现代物流业。促进工业物流提档升级，完善汽车物流中心服务功能，支持汽车物流企业做大做强，加快建设全国乃至全球汽车及零部件贸易名城；完成光彩物流中心二期4万平方米物流仓库和信息中心建设；围绕服务深圳工业园，抓紧编制现代工业物流园区规划。突破性发展农产品物流，支持中储粮物流项目建设，完成襄樊粮油及农副产品物流园一期工程，规划建设樊城农产品物流园区，初步形成全国重要的粮食物流节点。着力发展煤炭物流，在引资建设余家湖北煤南运物流园上实现突破，加快建设湖北重要的煤炭配送物流基地。做好综合性现代物流信息中心建设前期工作，尽快建立高效的物流公共信息与电子商务平台。

　　（五）努力转变发展方式，开创科学发展新局面

　　加快推进自主创新。把推进自主创新作为促进结构调整、转变发展方式的中心环节来抓。积极创建创新型城市，力争成为国家和省创新型城市试点。全面落实国家促进自主创新的财税金融措施，支持企业提高研发能力。加强创新平台建设，争创1-2个省级以上企业技术中心、2个省级工程技术研究中心，实施电机控制与电网优化装置、新材料、汽车零部件和摩擦材料等四个产业技术创新重大专项，带动相关行业技术升级。大力培植创新型企业，抓好22个总投资22亿元的自主创新项目建设，提高骨干企业创新能力，力争全年新认定高新技术企业25家以上，创建省级制造业信息化示范企业2-3家、省级科技创新型企业5家以上；支持高新区建设全国一流的科技孵化中心，孵化一批具有良好前景的创新型企业。大力推进人才强市战略，实施“隆中人才支持计划”，创造优越的环境，吸引各类高层次人才到襄樊工作；引导企业以股权、期权等方式吸引各类人才到企业创业，支持企业与高校和科研院所建立产学研联盟；重奖贡献突出的科技工作者，鼓励全社会踊跃创新。

　　加快发展“两型”产业。用足国家相关政策，实施积极的财税金融措施，鼓励企业自主研发和引进运用节能环保新技术，加快生产方式转型升级。重点推行工业废水闭路循环、粉煤灰和钢渣综合利用等循环经济生产方式，办好２个国家级、8个省级循环经济试点；促进所有开发区按照循环经济理念来发展，支持襄樊电厂火电循环经济园、谷城再生资源园和宜城大雁工业园建设，尽快形成全市“两型”产业发展示范区。

　　加快建设“两型社会”。严格落实节能减排目标责任制。强化节能减排措施，实施一批节能减排工程，确保实现节能减排目标。着力建设环境友好型社会。狠抓“一江五河”水污染防治和汉江沿岸生态环境综合治理，加快推进市区垃圾处理厂和４个市区污水处理项目建设，完成余家湖工业园污水处理厂新建和鱼粱洲污水处理厂扩建工程，确保县市污水处理厂全部建成投入运行。加快淘汰落后产能，严控新建高能耗、高污染项目，整治化工、印染、造纸等工业污染，强化以规模养殖基地为重点的农村环境污染治理。抓好退耕还林、植树造林，推进自然保护区和湿地公园建设，优化生态环境。着力建设节约型社会。推进工业能耗大户和建筑节能改造，规划开发一批节能环保型商住小区。抓住国土资源利用规划调整的有利时机，留足未来发展空间，加大建设用地报批力度，保障重点项目用地需求；落实最严格的耕地保护和节约用地制度，提高开发区新建项目投资强度和容积率，推进集约用地。

　　（六）着力实施民生工程，建设安定和谐新襄樊

　　始终把改善民生作为政府工作的出发点和落脚点，在确保财政收入持续稳定增长的同时，加大民生建设力度。

　　千方百计扩大就业，促进群众收入稳定增长。加快建立城乡一体的劳动力市场体系，统筹城乡就业工作。以促进困难群体、失业群体和大学生就业为重点，实施就业援助专项行动。大力实施“阳光工程”、“雨露计划”和“技能就业计划”，优先培训未升学的初中毕业生，提高城乡劳力就业能力。免费提供求职登记、职业指导、信息查询和职业介绍等就业服务，加大异地就业和本地就业推荐力度，及时为新建企业培训输送合格工人，引导社区吸纳就业，促进创业带动就业，把安排政府投资和确定重大项目与增加就业岗位结合起来，努力扩大就业渠道。千方百计拓宽农村劳力转移渠道，认真落实国家关于支持农民工创业的财税、金融措施，扶持一批农民工自主创业，大力发展回归经济。

　　加强保障体系建设，提高社会保障水平。加大城镇社会保险扩面核定、基金征缴和欠费清收力度，增强总体保障能力。按照省政府要求，做好养老保险省级统筹的各项准备工作；启动被征地农民养老保险，研究探索文化事业单位、国有林场、“小三场”、水利水管单位和村级主要干部养老保险的具体办法；完善城乡医疗保障体系，提高城镇居民医保、职工医保和新型农村合作医疗保障水平。加大社会救助力度，新增农村低保对象1万人、集中供养“五保”1000人，建成市孤残儿童养育康复中心；资助所有城乡低保户、五保户和重点优抚对象进入医疗保障体系；进一步提高低收入群体保障待遇，增加城乡低保补助，提高企业退休人员基本养老金和优抚对象生活补助标准；抓好78个村的整村推进扶贫工作，完成500个贫困户生态搬迁项目建设；大力发展慈善和养老事业，鼓励民间投资，启动市老年公寓建设，新增社会化养老床位1000张。

　　大力发展社会事业，着力改善公共服务。优先发展教育事业。巩固“普九”成果，完善义务教育经费保障机制，全面免除农村义务教育阶段寄宿制学生住宿费，以完善基础设施和提高教育质量为重点，进一步改善农村学校办学条件，抓好城区薄弱学校改造，推进区域义务教育均衡发展；完善贫困生资助体系，确保来襄经商、进城务工人员子女和留守、流动儿童接受义务教育；大力发展职业技术教育，整合职业教育资源，创建1-2所省级以上中等职业示范学校；提高普通高中教育质量；支持襄樊学院、襄樊职业技术学院和襄樊电大提高办学水平。大力发展卫生事业。按照国家和省统一部署，稳步推进医疗卫生改革；整合卫生资源，抓好城市社区卫生服务中心和县、乡、村三级医疗卫生机构基础设施建设，办好樊城省级社区卫生服务试点，提高基层卫生组织服务能力；加强艾滋病、结核病、血吸虫病等重大疾病预防控制,增加免费免疫接种的疫苗品种，预防相关传染病发生和流行。依法加强人口与计划生育工作，统筹解决人口问题，控制人口出生性别比，稳定低生育水平，降低人口出生缺陷率。巩固全国文明城市创建工作的成果，进一步提高市民素质和城乡文明程度。推进文化体制改革，增强文化事业发展活力；实施文化体育惠民工程，建设一批城市社区和农村文化体育设施，抓好20户以上自然村的“村村通”广播电视工程，推进市区数字化电视整体转换工作；深入开展送戏下乡、送电影下乡、全民健身和周末文化广场活动；狠抓以网吧监管和“扫黄打非”为重点的文化市场整治，净化文化环境。

　　切实解决群众关注的热点问题，全力维护社会安定。加强应急管理体系建设，增强应急处置能力；切实抓好各种自然灾害和重大事故测报、预防、应急、治理和恢复重建工作。深入推进“平安襄樊”建设，加强社会治安综合治理，严厉打击各类刑事犯罪，重拳惩处黑恶势力，全力维护社会稳定。继续支持驻襄解放军、武警部队、民兵预备役和人防工程建设，促进经济与国防事业协调发展。认真落实各级政府和企业安全生产的主体责任，建立健全乡镇安全生产监管网络，加强企业安全生产标准化管理，开展隐患排查和危险源监控，抓好高危行业和部位安全专项整治，防范安全生产事故。依法加强食品药品安全监管，实施食品质量安全市场准入制度，整顿食品作坊、饮食摊点卫生秩序，整治滥用食品添加剂和非食品原料的行为，确保食品安全。认真落实政府领导定期接访、下访和包案制度，畅通群众诉求表达渠道，强化联合督导和挂牌督办措施，切实把各种信访问题解决在基层。充分发挥人民团体、民主党派、民族宗教等各个方面在促进经济社会发展、构建和谐襄樊中的积极作用。

　　在做好以上各项工作的同时，市政府今年将集中力量办好关系民生的十件实事。

　　1、着力解决城市内涝问题，加快推进大李沟、南渠改造，完成闸口、马王庙、102、岘山、檀溪、前进路、大庆路、迎旭门、汉江路、樊西等十片区重点路段排水管网改造，更新改造东门、城南、闸口、岘山、丹江路、迎旭门、汉江、清沙厂、六化建等九座泵站，提升城市排水能力。

　　2、实现医疗保险（职工医保、新型农村合作医疗、城镇医保）全覆盖，实现新型农村合作医疗和城镇医保愿保尽保。

　　3、培训城乡劳力5万人，转移农村劳力8万人；开发公益性岗位3000个，新增城镇就业人员8.6万人。

　　4、实施保障性安居工程，筹集廉租房源3100套，改造棚户区3万平方米，改造农村危房1000户。

　　5、解决农村40万人的饮水安全问题，增加沼气用户4万个。

　　6、建设通村公路400公里、农村客运站6个、招呼站200个，基本实现村村通。

　　7、基本完成市区24所薄弱学校硬件改造，努力提高教育教学质量，区级以上骨干教师比例达到10%以上，初中、小学新生在学区内入学率分别达到70%和80%以上。

　　8、加强城乡文化体育设施建设，完成10个乡镇综合文化站、400个农家书屋和10个城市社区流动图书站建设，为90个村、20个城市社区新建体育设施。

　　9、为困难群众提供法律援助，全年办理法律援助案件2000件以上。

　　10、开工建设城市生活垃圾集中处理焚烧发电工程，实现市区垃圾无害化处理。

　　三、关于政府自身建设

　　做好今年的政府工作，必须切实加强市政府及其各部门自身建设，转变工作作风，提高行政水平，凝聚促进发展的强大合力。

　　深入学习和实践科学发展观，提高领导科学发展的能力。认真按照省委和市委的统一部署，组织政府及其各部门领导班子和领导干部，深入学习实践科学发展观，努力提高领导科学发展的能力。在深入调查研究、认真分析查摆的基础上，以科学发展观指导和改进政府工作。着眼于解决发展不够的问题，深刻认识科学发展观的第一要义，坚定不移地把发展作为第一要务，更加自觉、更加坚定地牢牢扭住经济建设这个中心，抢抓机遇、大干快上，奋力促进全市经济跨越式发展；着眼于实现好、维护好、发展好最广大人民群众的根本利益，努力使贯彻落实科学发展观的过程，成为不断提高人民生活水平的过程。着眼于解决当前经济社会发展中的突出矛盾和问题，坚持解放思想，实事求是，把发展水平作为衡量思想解放程度的主要标准，把问题解决程度作为衡量领导科学发展能力的主要标志，促进各级各部门在实践中解放思想，在解放思想中统一思想，自觉把改革创新精神贯彻到政府工作各个环节，突破各种束缚发展的条条框框，用改革的办法、创新的举措开创科学发展新局面。

　　创新体制机制，努力提高政府效能。按照中央和省统一部署，启动市、县政府机构改革，积极稳妥地推进事业单位人事制度改革，加快转变政府职能，着力构建充满活力、富有效率、更加开放、有利于促进科学发展的体制机制。牢固树立“产业第一、企业优先”的思想，深入推进“阳光新政”，为企业提供“保姆式”服务。加强各级行政服务中心建设，提升综合服务功能；按照所有工作程序和方式必须服从和服务于发展的要求，清理各类影响招商引资、项目建设的政策规定，再造优质高效的服务流程；大力推行电子政务和网上审批，压缩审批时限，推进审批提速。建立科学的行政服务评价机制，严肃查处经济环境投诉案件，促进经济环境持续改善。建立强有力的工作推进机制，实行重点工作一名领导、一个专班、一抓到底；进一步完善政府工作目标管理，设计更加科学的考核指标体系，建立健全目标考核结果与奖惩挂钩制度，强化表彰奖励和行政问责措施，强力推进政府工作。

　　推进依法行政，切实规范政府行为。进一步完善决策机制，规范决策程序，认真执行重大事项专家论证、公开听证制度，主动听取民主党派、工商联、无党派人士和专家学者对政府工作的意见和建议，促进政府决策更加符合科学发展观的要求。认真执行人民代表大会及其常委会各项决议，自觉接受人民代表大会的法律监督，主动加强与人民政协的联系，认真办理人大代表议案、建议和政协委员提案。加强城市社区和村民自治，新建100个村务公开、民主管理示范村（居）委会。加强政府法制建设，狠抓依法行政考核，促进各级政府及其工作部门提高依法行政水平；规范行政执法程序和行政处罚自由裁量权，严格执行执法责任制、错案和执法过错追究制，切实解决有法不依、执法不公、执法扰民等群众反映强烈的问题。

　　坚持从严治政，着力建设廉洁政府。深入落实党风廉政建设责任制，加强对政府工作人员的教育和管理，坚决支持纪检监察机关查处腐败案件。狠抓源头治腐，严肃财经纪律，加强财政预算管理，压缩一般性开支，规范公务支出和公车使用，努力降低行政成本；深入开展行政事业单位执行收费政策、使用专项资金以及出租资产情况专项检查，彻底整治“小金库”；推进政务公开，加强对重大决策、行政执法、政府投资、财政资金运行、产权交易、土地转让、工程招投标、经济适用房和廉租房分配等重点领域的监督，防止暗箱操作、滋生腐败，树立廉洁政府的好形象。

　　各位代表，新的一年，政府工作任务繁重而艰巨。我们将紧紧依靠省委、省政府和市委的坚强领导，解放思想，抢抓机遇，迎难而上，创新实干，为加快推进全市经济社会跨越式发展，建设名副其实的省域副中心城市而努力奋斗！
